# Composite Drug Delivery System Based on Amorphous Calcium Phosphate–Chitosan: An Efficient Antimicrobial Platform for Extended Release of Tetracycline

**DOI:** 10.3390/pharmaceutics13101659

**Published:** 2021-10-11

**Authors:** Anita Ioana Visan, Carmen Ristoscu, Gianina Popescu-Pelin, Mihai Sopronyi, Consuela Elena Matei, Gabriel Socol, Mariana Carmen Chifiriuc, Coralia Bleotu, David Grossin, Fabien Brouillet, Sylvain Le Grill, Ghislaine Bertrand, Irina Zgura, Rodica Cristescu, Ion N. Mihailescu

**Affiliations:** 1 National Institute for Laser, Plasma and Radiation Physics, 077125 Magurele, Romania; carmen.ristoscu@inflpr.ro (C.R.); gianina.popescu@inflpr.ro (G.P.-P.); mihai.sopronyi@inflpr.ro (M.S.); consuela.matei@inflpr.ro (C.E.M.); gabriel.socol@inflpr.ro (G.S.); rodica.cristescu@inflpr.ro (R.C.); 2 Department of Microbiology, Faculty of Biology, University of Bucharest, 060101 Bucharest, Romania; carmen.chifiriuc@gmail.com; 3 Earth, Environmental and Life Sciences Division, Research Institute of the University of Bucharest, 050567 Bucharest, Romania; cbleotu@yahoo.com; 4 Stefan S. Nicolau Institute of Virology, 285 Mihai Bravu Ave, Sect. 3, PO 77, P.O. Box 201, Bucharest 030304, Romania; 5 CIRIMAT, CNRS, INP-ENSIACET, Université de Toulouse, 4 allée Emile Monso, 31030 Toulouse, France; david.grossin@ensiacet.fr (D.G.); ghislaine.bertrand@ensiacet.fr (G.B.); 6 CIRIMAT, CNRS, Université Toulouse 3-Paul Sabatier, 35 Chemin des Maraîchers, CEDEX 9, 31062 Toulouse, France; fabien.brouillet@univ-tlse3.fr (F.B.); sylvainlegrill@gmail.com (S.L.G.); 7 National Institute of Materials Physics, 077125 Magurele, Romania; irina.zgura@infim.ro

**Keywords:** prolonged drug release, antimicrobial films, implant coatings, nosocomial infection prevention, amorphous calcium phosphate, chitosan, tetracycline, matrix-assisted pulsed laser evaporation (MAPLE)

## Abstract

One major warning emerging during the first worldwide combat against healthcare-associated infections concerns the key role of the surface in the storage and transfer of the virus. Our study is based on the laser coating of surfaces with an inorganic/organic composite mixture of amorphous calcium phosphate–chitosan–tetracycline that is able to fight against infectious agents, but also capable of preserving its activity for a prolonged time, up to several days. The extended release in simulated fluids of the composite mixture containing the drug (tetracycline) was demonstrated by mass loss and UV–VIS investigations. The drug release profile from our composite coatings proceeds via two stages: an initial burst release (during the first hours), followed by a slower evolution active for the next 72 h, and probably more. Optimized coatings strongly inhibit the growth of tested bacteria (*Enterococcus faecalis* and *Escherichia coli*), while the drug incorporation has no impact on the in vitro composite’s cytotoxicity, the coatings proving an excellent biocompatibility sustaining the normal development of MG63 bone-like cells. One may, therefore, consider that the proposed coatings’ composition can open the prospective of a new generation of antimicrobial coatings for implants, but also for nosocomial and other large area contamination prevention.

## 1. Introduction

The large-scale development of implants in different medical fields is, unfortunately, restricted by a growing number of related infections [1]. The associated severe complications often lead to prolonged excruciating pain, increased costs, and eventually implant loss and mandatory revision [2,3]. Nosocomial infections constitute a major problem with high incidence and high morbidity and mortality rates in worldwide health institutions. Enhanced prevention and control of nosocomial infections is therefore a current strategic priority [3].

Various approaches aiming to reduce the microbial load and promoting the preservation of a microbe-free environment have therefore been implemented to counteract infection risks [4]. A major strategy in implant development resorbs to the design of coatings consisting of antibiotics-based composite material to cover the surface of metal implants (with appropriate mechanical properties) to ensure a high, long term antimicrobial efficacy [5,6]. A more effective strategy is to construct smart controlled delivery systems using compounds with simultaneous and complimentary action against infectants. These complex systems should allow precise control of both local concentration and release rate in order to provide efficient care therapies [7,8]. In the last few years, the design and methodology to obtain such systems have developed considerably. Different coating methods (e.g., cold spraying [9], dip coating [10], electrochemical methods [11], electrophoretic deposition [12], magnetron sputtering [13], photochemical oxidation [14], and matrix-assisted pulsed laser evaporation (MAPLE) [15,16,17]) were applied for incorporating various antibiotics (e.g., gentamicin [18], vancomycin [19], tobramycin [20], rifampicin [21,22], or tetracycline hydrochloride [23,24]) onto bioactive surfaces.

The MAPLE technique was our option for the fabrication of antimicrobial coatings, suitable for the prevention of inherent surface infections by local, sustained antibiotic delivery. It is well known that MAPLE secures the stoichiometry preservation of delicate compounds after transfer, ensures a high accuracy of control of deposition thickness [25,26], ensures minimum contamination, and supposes low production costs and biomaterial consumption [27,28]. That is why MAPLE rapidly extended from medical implant manufacturing to tissue engineering applications [29].

We propose herewith a new dual antimicrobial approach based upon an antibiotic (tetracycline (TC)) potentiated by a natural antimicrobial agent (chitosan (CHT)) to stabilize the antimicrobial activity, prevent drug resistance [30], and regenerate surrounding living tissues. To improve impact, the composition was completed with amorphous calcium phosphate (ACP) recognized for the capacity to promote bone growth and healing.

ACP is often chosen as a precursor [31] to prepare bioactive biomaterials due to its high reactivity and facile conversion into poorly crystalline apatite analogous to bone mineral phase [32]. Moreover, ACP was previously exploited, especially to prepare coatings with enhanced adhesion and bioactivity [33,34]. Furthermore, ACP exhibits a better solubility than other crystalline Calcium Phosphates (CaPs) and the ability to promote osteoblast adhesion and osteoconductivity [35]. Efficient bone healing is expected via bone homeostasis induction and mineral formation promoted by the proposed carrier under physiological conditions [36]. ACP provides mechanical strength to bone and serves as a storage medium for mineral ions (mainly calcium and phosphate groups). These ions play a critical role in the biomineralization of bone [34,35]. During the biomineralization of natural bone tissue, the initially formed solid phase is ACP, which further converts into crystalline apatite within the organic matrix [37,38]. ACP is thermodynamically unstable and tends to spontaneously transform to crystalline CaP [39]. Such instability and easy transformation towards the crystalline phase have great biological relevance. Specifically, the ACP’s role in the biomineralization of the matrix vesicle demonstrates its importance as a pivotal intermediate in skeletal calcification [39]. Due to its significant chemical and structural similarities to calcified tissues, as well as good biocompatibility and bioresorbability, ACP is a very promising candidate for the manufacture of artificial bone [40]. ACP has also been considered for drug delivery due to its high capacity for drug loading and controlled release [35].

The apatitic-based substrates can serve as a strong support for a wide range of therapeutic agents [37,38,41]. Recent studies [39] pointed out that both the adsorption and release of bioactive drug molecules are strongly dependent on the chemical and structural characteristics of the apatitic-based substrates, besides the chemical properties of drug molecules [42]. The drug release rate can therefore be finely tuned by the proper choice of the drug incorporating matrix [43,44,45,46].

In this study, the highly biodegradable, non-toxic, and biocompatible CHT was selected as antimicrobial agent [47] due to the potential applications as implant surface scaffolds and drug delivery systems. CHT has a similar chemical structure to the organic phase of bone (i.e., glycosaminoglycan, the prevalent extracellular matrix of the bone and cartilage). Thus, the combination of the antibiotic (i.e., TC) with a non-antibiotic adjuvant (i.e., CHT) should potentiate the efficient prevention of infections due to multiplication of antimicrobial mechanisms. Moreover, one expects that the addition of CHT allows for a smaller consumption of the antibiotic and thus minimizes the risks of drug resistance.

There are some reports dealing with the combined antimicrobial effect of antibiotics and CHT (or its derivatives) [48,49] or ways to improve some of its properties through functionalization with TiO_2_ [50]. It was previously shown [51,52] that the antimicrobial activity of bioactive metastable calcium phosphate, such as nanocrystalline biomimetic apatite–chitosan mixture, can be monitored by the chitosan content that controls, at the same time, the adherence to the deposition substrate.

TC was assigned as an antibiotic due to its recognized efficiency against microbes (i.e., bacteria (both Gram-positive and Gram-negative) and fungi and its common use in the treatment and prevention of infections. Also, TC exhibits a large binding affinity to bone, namely to calcium ions [53].

Summarizing the information presented above, the aim of this paper is to explore the potential of the MAPLE deposition method in the fabrication of functionalized surfaces for medical use whenever controlled drug release is intended. In this context, the main objectives within our study are: (i) to estimate the drug release from composite coatings under simulated (body) conditions, (ii) to determine the in vitro antimicrobial agent efficiency, and (iii) to evaluate drug cytotoxicity against host cells.

## 2. Materials and Methods

### 2.1. Materials

We used an ACP powder synthetized after a recipe describe elsewhere with a 1.3 Ca/P ratio [54]. Commercial powders of CHT (low molecular weight, 50–190 kDa) and TC (tetracycline phosphate salt, CAS No. 1336-20-5) were purchased from Sigma Aldrich (Sigma-Aldrich Chimie S.a.r.l., Saint Quentin Fallavier, France). All chemical reagents were of analytical purity and used with no further purification.

Composite powder of ACP/CHT/TC was obtained after drying a solution. Targets used for MAPLE deposition were prepared according to the protocol described in References [36,52]. The proportions of the ACP, CHT, and TC in the composite powder were 64.8%, 35%, and 0.2% (wt), respectively, though it should be mentioned that the composite was prepared as it was redispersed.

The bio-reagents were purchased either from Sigma–Aldrich: (paraformaldehyde, hexamethyldisilazane (HMDS), non-essential amino-acids (NEAA)), or Merck (Biochrom A.G., Berlin, Germany): phosphate buffered saline (PBS), fetal bovine serum (FBS), Eagle’s Minimum Essential Medium (MEM), and L-glutamine and penicillin/streptomycin mixture (P/S).

MTS kit (CellTiter96^®^ Aqueous One Solution Cell Proliferation Assay) used for the cellular viability tests was from Promega (Promega Corporation, Madison, WI, USA). The stains for immunofluorescence studies (Texas Red^TM^–Phalloidin solution and Hoechst^®^ 33,342) were acquired from Invitrogen (Thermo Fisher Scientific, Waltham, MA, USA). Samples were then stained with propidium iodide (PI) and fluorescein diacetate (FDA).

Osteoblast-like cells (MG63) (ATCC crl-1427) and microbial pathogens, namely *Escherichia coli* (*E. coli*, ATCC 25922) and *Enterococcus faecalis* (*E. faecalis*, ATCC 8739) were procured from American Type Culture Collection (ATCC^®^, Manassas, VA, USA).

### 2.2. MAPLE Deposition

**Figure 1:**
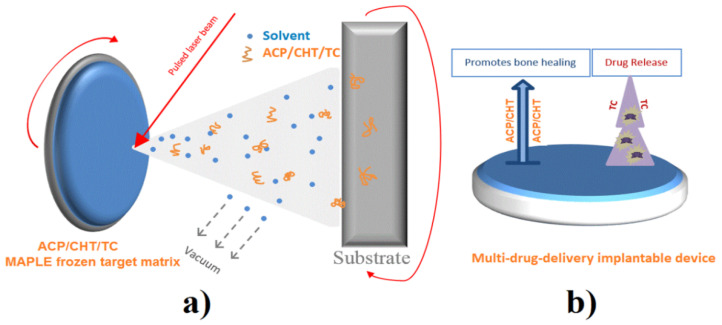
(**a**) MAPLE experimental scheme and (**b**) multi-drug delivery implantable device.

The MAPLE set-up used in experiments is schematically presented in Figure 1.

Appropriate amounts of the ACP/CHT/TC powder mixture were dispersed in deionized water (0.077 g powder in 5 mL water) to prepare target solutions with an ACP concentration of 1% *w/w*. The experiments were conducted in the dark, as a protection from TC photosensibility. The Berzelius recipient was therefore wrapped in an aluminum thin foil. The solution was homogenized by magnetic stirring and then frozen in a copper holder for 15 min in the presence of liquid nitrogen (LN). The holder with the “icy” target was placed in contact with a cooler kept at low temperature by LN circulation during experiment. Both target and substrate were rotated by 50 rpm during deposition, in order to avoid piercing the target and ensure a better uniformity of the growing films (Figure 1). The deposited composite films were further denoted ACP/CHT/TC.

An excimer laser source KrF* (COMPexPro 205 F, λ = 248 nm, pulse duration τ = 25 ns, and ν = 10 Hz-Lambda Physics-Coherent, Göttingen, Germany) was used for target evaporation and film deposition. A beam homogenizer was intercalated between the laser source and the deposition chamber to improve the energy distribution of the laser spot on the target surface.

10 mm^2^ glass and double-side polished (100) Si slides as well as grade 4 titanium disks (PDF 00-044-1294 Card, alpha phase) of 12 mm diameter and 0.2 mm thickness were used as deposition substrates for further investigations. The separation distance of the target–substrate was set at 5 cm. Substrates were placed parallel to the target and maintained at room temperature (RT) during deposition. Prior to deposition, substrates were subsequently cleaned in an ultrasonic bath in acetone, ethanol, and deionized water and blow-dried with 99% pure N_2_. For comparison, controls were prepared by dropping solutions on (100) Si wafers.

Deposition was optimized against incident laser fluence and pressure inside the working chamber. The best combination was found for 0.5 J/cm^2^ and 1 Pa, for which a congruent (no loss) transfer was ensured from target to substrate. A series of 25,000 subsequent laser pulses was applied for the deposition of each bunch of samples.

### 2.3. Physico-Chemical Characterization

#### 2.3.1. Coating’s Morphology

Coating’s morphology has been examined by Scanning Electron Microscopy (SEM), conducted with an ApreoS instrument (Thermo Fisher Scientific, Waltham, MA, USA), working in high vacuum, with an acceleration voltage of 10 kV, under secondary electron mode.

#### 2.3.2. IR Active Groups

IR active groups of ACP/CHT/TC composite from samples deposited onto IR transparent (Si (100)) was monitored by **Fourier transform infrared (FTIR) spectroscopy**. Investigations were carried out with a Shimadzu IRTracer-100 spectrophotometer (Shimadzu Europa GmbH, Duisburg, Germany), operating in absorbance mode (for original composite powder and MAPLE coating) and transmittance (for degraded coating) within the (400–4000) cm^−1^ range, with a 4 cm^−1^ resolution. Each IR spectrum was obtained as an average of 50 individual scans.

#### 2.3.3. X-ray Diffraction (XRD)

The crystallinity of deposited coatings was assessed by **X-ray Diffraction (XRD)** using a X’Pert Pro MPD Bruker D8 Advance diffractometer from Panalytical (Bruker AXS, Karlsruhe, Germany), equipped with a Cu target X-ray tube, (λ = 1.5406 Å) set to work in Bragg–Brentano geometry in parallel beam setting. The incidence angle was set at 2°, while the scattered intensity was acquisitioned within the range of (10–50)° (2θ), with 0.04° step size and 6 s per step.

#### 2.3.4. Static Contact Angle (CA)

Static contact angle (CA) measurements were performed by the goniometric method at RT with a Drop Shape Analysis System, model DSA100, equipped with DSA3^®^ software (Kruss GmbH, Hamburg, Germany) using either deionized water or diiodomethane as standard solutions. A medium measuring 1 μL was dropped under the control on the sample surface, constantly preserving both the droplet volume and dropping distance. The droplet image was acquired under a 2° angle with respect to the surface of the examined sample. Solid surface free energy (SFE) calculations were performed based upon the concept of polar and dispersion components (Owens–Wendt approximation) [55,56,57]. Duplicate experiments were carried out in each case and results are presented as mean value ± standard deviation (SD).

#### 2.3.5. Drug Release

Drug release of TC entrapped in the composite coatings was investigated in dynamic regime in simulated body fluid (SBF) and prepared according to the well-known Kokubo recipe [58] (pH = 7.45 at 37 °C). An injection multichannel degradation reactor that simulates the flow of blood flux inside human tissues (with (1.31 ± 0.04) mL/min flow rate) was used, according to the protocol described in Reference [26]. Calibrated hoses of 1.6 mm inner diameter (67 cm long) circulated 4 mL SBF through each channel containing twin samples.

#### 2.3.6. Mass Loss Evaluation

Mass loss evaluation of composite samples in each channel of the reactor was carried out at different dynamic testing times, *t*, of 2, 4, 6, 8, 12, 24, 48, and 72 h.

Mass loss, Δ*m*(*t*), was evaluated as the difference between the initial weight of the Ti substrate with as-deposited film, *M*_0_, and residual weight of film and substrate after immersion in SBF for a specific time, *M*(*t*):

Δ*m*(*t*) = *M*_0_ − *M*(*t*) (1)

The degradation loss percentage of the composite mixture (containing antibiotic), *p*, was calculated as:

*p* = [(*M*_0_ − *M*(*t*))/*M*_0_] × 100% (2)

All mass measurements were performed with a Radwag MYA 0.8/3.3Y analytical microbalance (Radwag^®^ Wagi Elektroniczne, Radom, Poland).

In vitro release kinetics of TC were monitored by **UV–Vis absorption spectroscopy** (Evolution 220 Spectrophotometer, ThermoFisher Scientific, Shanghai, China), using the SBF solution collected from the bioreactor cells at different times (2, 4, 6, 8, 12, 24, 48, and 72 h).

#### 2.3.7. pH Evolution of SBF

The pH evolution of SBF solutions with released composite components from MAPLE films was monitored after biodegradation with a Consort pH-meter model C861 (Consort, Turnhout, Belgium).

### 2.4. Microbiological Assay

#### 2.4.1. Biocompatibility

Biocompatibility was evaluated in vitro using human bone osteosarcoma (MG63, ATCC^®^ CRL-1427™) cells. Both morphology and viability were investigated for cells growing on the MAPLE thin films deposited on Ti substrate. For microscopic observation, the specimens were placed in 24-well plates, exposing the coated sample surface. Then, 5 × 10^5^ MG63 cells were added in Dulbecco’s Modified Eagle’s Medium (Dulbecco’s MEM) supplemented with 10% fetal bovine serum and 1% essential amino acids. The plates were preserved for 24 h at 37 °C in 5% CO_2_. Samples were then stained with propidium iodide (PI) and fluorescein diacetate (FDA), and with PI and Hoechst (H), in order to differentiate among dead (PI) and viable cells (FDA, H). They were immediately visualized and subsequently photographed in fluorescence with a Leica DFC450C microscope (Leica Microsystems Lt, Heerbrugg, Switzerland).

For the cellular cycle assay, MG63 cells were cultivated into a 1640 Roswell Park Memorial Institute (RPMI) medium (GIBCO^TM^ by Thermo Fisher Scientific Inc, NY, USA) supplemented with 10% heat-inactivated bovine serum and penicillin/streptomycin and incubated at 37 °C in 5% CO_2_ for 24 h. Monolayers were then harvested, washed with phosphate buffered saline (PBS), fixed in 70% cold ethanol, and incubated overnight at −20 °C. Each sample was washed in PBS, treated with 100 μg/mL RNase A for 15 min, and stained with 10 μg/mL PI by incubation at 3 °C for 1 h. Events acquisition was carried out with an Epics Beckman Coulter^®^ flow cytometer (Beckman Coulter Inc., Nyon, Switzerland). The data obtained were analyzed using the FlowJo^TM^ software (FlowJo LLC, Ashland, OR, USA) and expressed as fractions of cells under different cell cycle phases.

#### 2.4.2. Antimicrobial Biofilm Activity

The antimicrobial biofilm activity of samples was investigated against *Enterococcus* *faecalis* ATCC 29,212 and *Escherichia* *coli* ATCC 8739 strains. The samples were first UV sterilized for 30 min. A quantity of 20 µL microbial suspension of 0.5 McFarland density prepared from fresh cultures developed on tryptic soy agar (TSA) was spread over the sample’s surface and incubated at 37 °C in a humid atmosphere for 15 and 30 min and 1, 4, 6, 24, and 48 h. After incubation, the samples were suspended in 5 mL sterile saline and vortexed vigorously to re-suspend the adherent bacteria. Serial ten-fold dilutions were then carried out from the recovered suspensions, spread on solid medium in triplicate spots of 10 µL each, and the viable cell counts (VCCs) were next performed and expressed as colony forming units (CFU)/mL. The two-way ANOVA Bonferroni test was conducted using GraphPad Prism version 5.00 for Windows, GraphPad Software (La Jolla, CA, USA, www.graphpad.com, accessed on 8 October 2021).

## 3. Results

### 3.1. Physico-Chemical Investigations

#### 3.1.1. SEM

**Figure 2:**
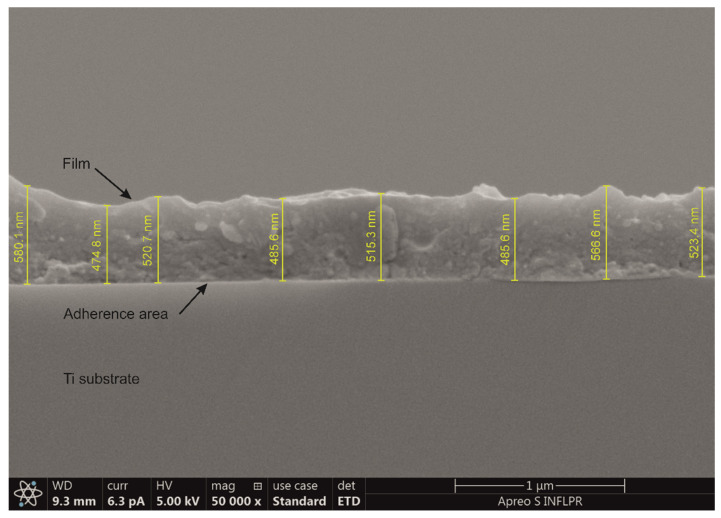
Typical cross-section SEM image of as-deposited composite coating.

The typical cross-section image in Figure 2 discloses a compact, quite uniform aspect of the coating, supporting a good adherence of the deposited film to the substrate. An average thickness of ~500 nm was measured corresponding to a deposition rate of 0.02 Å/pulse.

The actual behavior of the composite coatings after implantation was simulated by controlled immersion in SBF. After immersion, the samples were carefully dried in ambient air and then in vacuum at a residual pressure of 10^−2^ Pa. The films were investigated by the same physico-chemical methods, after different times of immersion.

**Figure 3:**
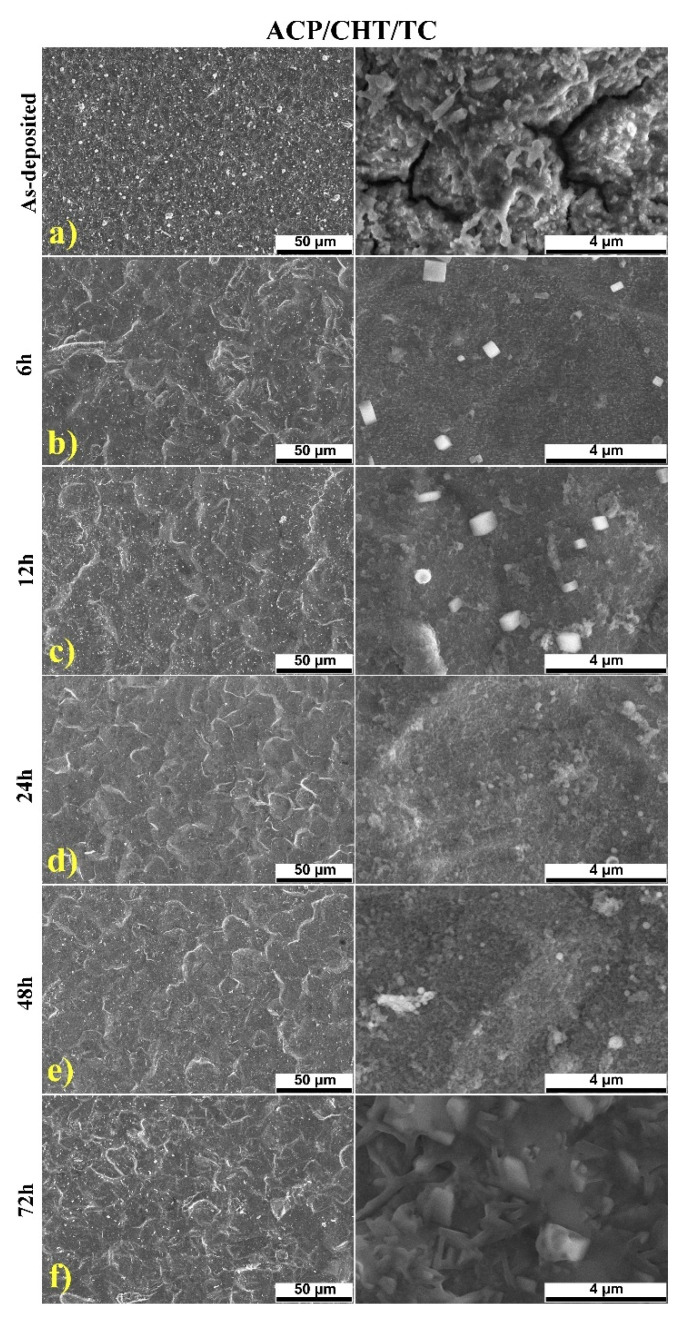
Typical SEM micrographs of: as-deposited ACP/CHT/TC coating (**a**), and after 6 (**b**), 12 (**c**), 24 (**d**), 48 (**e**), and 72 (**f**) hours degradation in SBF (left column 50 μm magnification and right column 4 μm magnification).

For comparison, in Figure 3 we collected typical SEM micrographs of the surface of composite films freshly deposited (a) and after different immersion times in SBF (b–f). By careful inspection of Figure 3a, the following observations are in order:

1. Particles of different size, up to several μm, are present on the surface of the deposited films. They are assembled in a rather smooth morphology, characteristic to MAPLE ACP coatings [36].
2. Films are quite homogenous and exhibit few microcracks, which do not affect the coating’s toughness or, notably, the adherence to the substrate. In our opinion, this is a beneficial characteristic of this composite, allowing the formation of a CHT continuous matrix where ACP and TC are finely entrapped, favoring a high adherence to the substrate [36,50].

SEM examination showed a uniformity improvement trend with the immersion time in SBF. The initial aspect of the film gradually changed to the finer one with few small pores (of μm-size). A positive role of these pores is expected for prolonged drug delivery [59].

The cracks visible after deposition (Figure 3a) disappeared and were replaced by submicronic paralelipiped crystals (Figure 3b,c), most probably of NaCl from SBF, since Na is not present in any other chemical formula of the employed substances and all the maneuvers were cautiously executed to prevent any contamination. Their size and number increased with immersion time (Figure 3b,c) only to decrease and disappear completely after 24 h (Figure 3d). The structure looked more eroded after 72 h of immersion in SBF (Figure 3e).

The comparison between the content of as-deposited films vs. films after prolonged degradation in SBF (EDS data presented as Figure S1 Supplementary Material) revealed the predominance of elements from basic components (Ca, P) of the composite mixture, but also traces (Na, Mg, Cl) originating from SBF.

#### 3.1.2. FTIR

**Figure 4:**
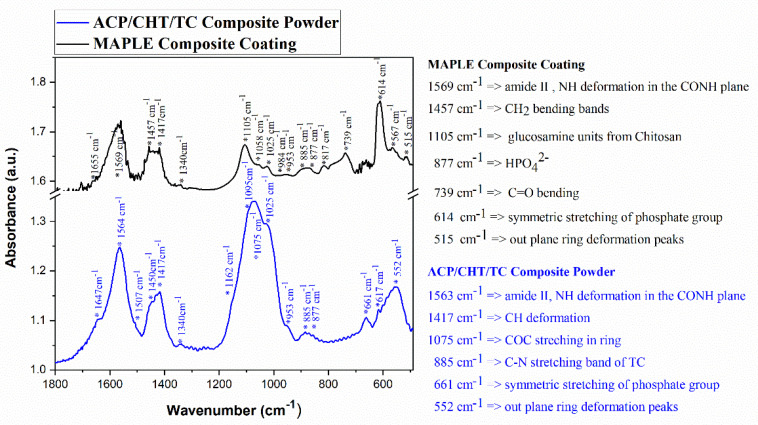
Typical FTIR spectra of composite ACP/CHT/TC powder (blue) and freshly deposited composite film (black). Significant peaks are mentioned.

Characteristic FTIR spectra of raw composite powder (blue) vs. deposited composite film (black) were displayed in Figure 4. An important observation is the obvious similarity between composite powder vs. film spectra. The predominant peaks in the two spectra are identical (for convenience, they were mentioned in the right-hand side of Figure 4).

Thus, peaks in as-deposited film spectrum at 877 (HPO_4_^2−^), 614 (ν_4_PO_4_), and 515 (out plane ring deformation peaks ν_4_PO_4_) cm^−1^ are also visible in the composite powder spectrum as well, at 616 and 552 cm^−1^, respectively [60].

Regarding the ACP component, one notices the presence in MAPLE film vs. composite powder of the following peaks: 1569 vs. 1564 (CO); 1457 vs. 1450 (CO_3_); 1417 vs. 1422 (CO_3_); and 1105 vs. 1075 (ν_3_PO_4_, shoulder) [61]. The low resolution of these peaks is characteristic of ACP [62]. IR spectroscopy also made it possible to highlight the characteristic bands of water around 3500 cm^−1^ and 1650 cm^−1^ [63]. CHT presents specific bands for the constitutive groups—the amide: 1655, 1560, and 1325 (respectively C=O, NH, CN), amine: 1594, and CHT skeleton: 1453, 1421, 1381 (alcohols) and 1162, 1075, and 984 cm^−1^. On the other hand, it is worth mentioning that the CHT characteristic features could be overlapped with apatite bands [64]. Characteristic bands of the CHT skeleton at 1162, 1075, and 984 cm^−1^ partially overlap with the ACP bands at 1095 and 1058 (PO_4_), and 877 cm^−1^ (HPO_4_). In our opinion, this effect is boosted by the predominant amorphous nature and rather low thickness of the deposited analyzed layer.

It should be also stressed that the broad bands of the composite coating overlapped, to a great extent, with the major absorbance features of TC. The band peaking at 1452 cm^−1^ in pure TC powder [65] and assigned to asymmetrical CH_3_ bending/ring C–C stretching is close to the peak at 1417 cm^−1^, in case of composite powder, and at 1452 cm^−1^ in film. The C–C out-of-plane bending centered at 574 cm^−1^ in pure TC powder is present at 552 and 515 cm^−1^ in composite powder and deposited film, respectively. Finally, the C–N stretching band centered at 885 cm^−1^ in pure TC powder is peaking at the same wavelength in the case of both composite powder and deposited film [66].

**Figure 5:**
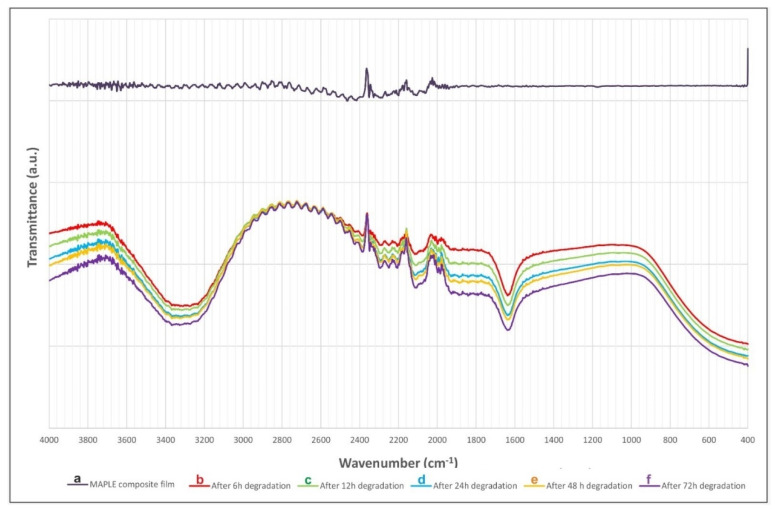
ATR–FTIR spectra of MAPLE coating: as-deposited (**a**) and after different degradation time intervals in SBF at 37 °C: after 6 (**b**), 12 (**c**), 24 (**d**), 48 (**e**), and 72 (**f**) hours, respectively.

The substance still present on the substrate after degradation is compositionally similar to raw powders and as-deposited films (Figure 5). One notices that the characteristics bands of OH stretching and bending of water around 3300 and 1600 cm^−1^ relatively increase with the immersion time.

#### 3.1.3. XRD

**Figure 6:**
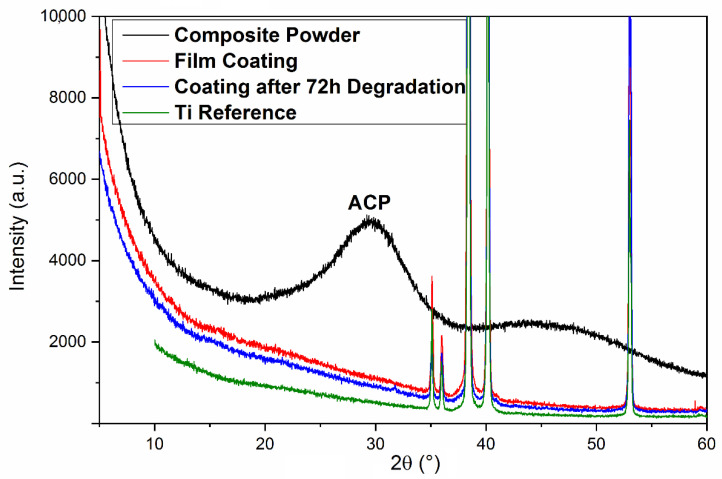
XRD diffractograms of: Ti substrate (green); ACP/CHT/TC composite powder (black); ACP/CHT/TC as-deposited film (red); and MAPLE composite coating after 72 h of immersion in SBF at 37 °C (blue).

The XRD diffractogram of a freshly deposited composite film on Ti substrate acquired within the (3–57)° range is given in Figure 6, together with that of one of the most degraded films. For reference, the XRD pattern of the bare Ti substrate and composite powder are presented.

From Figure 6, one notices the predominance of Ti diffractions peaks over the rather low background of species in the film. Thus, characteristic Ti peaks (ICDD: 00-044-1294) are visible at 35° (100), 38.4° (002), 40.23° (101), and 53° (102) planes A low intensity “hump” between 25 and 36°, integrating the signals from ACP (the broad peak centered around 30°), was observed. The halo, however, is visible only in the case of composite powders, due to the combined effects of the prevalent amorphous state of the deposited compounds and the low thickness of as-deposited or degraded thin films. As expected, apatite, CHT, and TC phases seem to exhibit an amorphous predominant nature [65].

The XRD pattern recorded after 72 h of immersion in SBF evidenced, as supposed, a rather amorphous character of the degraded film.

#### 3.1.4. Wettability

**Figure 7:**
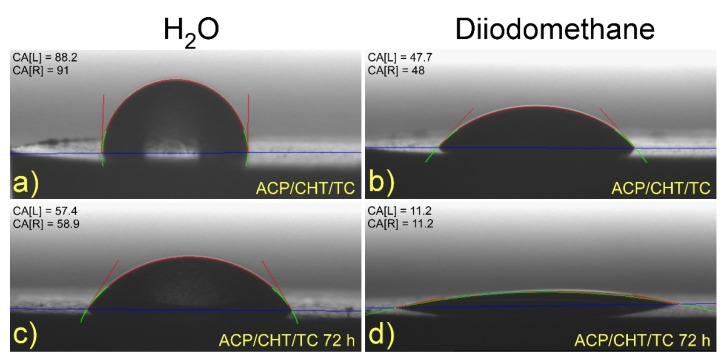
Typical images of (**a**,**c**) water and (**b**,**d**) diiodomethane droplets on as-deposited composites films (**a**,**b**) and after 72 h of degradation in SBF (**c**,**d**).

Typical images are given in Figure 7 of as-deposited composite films on Ti substrate, with deionized water (a) and diiodomethane (b) droplets. For convenience, similar images of biodegraded films after 72 h immersion in SBF were also displayed in Figure 7 for deionized water (c) and diiodomethane (d).

An increase in the hydrophilicity of film is noticed after immersion in SBF (compare Figure 7a,b to Figure 7c,d).

**Figure 8:**
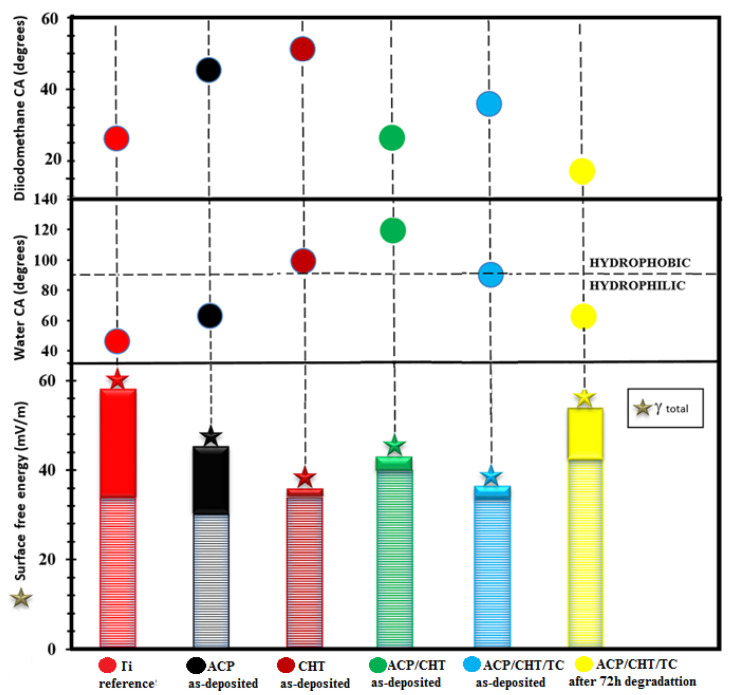
The water and diiodomethane contact angle (CA) and surface free energy (SFE) recorded for the bare Ti substrate and ACP, CHT, ACP/CHT, and ACP/CHT/TC as-deposited coatings vs. ACP/CHT/TC coating after 72 h of degradation in SBF (ᵧd—cross-hatched region, ᵧp—solid region).

The measured contact angle values were collected in Figure 8 in the case of composite film vs. Ti as reference and ACP, CHT, and ACP/CHT as base components. The drop profiles were fitted with a circular, or second-degree, polynomial equation in order to obtain the contact angle values.

After examination of Figure 8 the following observations are in order:

1. As-deposited films
  - Ti is highly hydrophilic: 47° CA in deionized water/28° in diiodomethane and 56.2 mN/m SFE.
  - A similar behavior is observed for ACP: 63°/42° and 44 mN/m.
  - CHT is slightly hydrophobic: 98°/51° and 34 mN/m.
  - ACP/CHT is super hydrophobic: 119°/50° and 42 mN/m.

The composite film ACP/CHT/TC is close to the transition border [57] from hydrophilic–hydrophobic, but on the hydrophilic side: 89°/47.8° and 35.5 mN/m. It seems that TC inclusion “pushes” the mixture from super hydrophobic to slightly hydrophilic [67]. This behavior is confirmed by SEM micrographs in which as-deposited films exhibit an improved morphology in the case of ACP/CHT as against ACP/CHT/TC films. Similar features were reported in References [68,69].

2. After 72 h of immersion in SBF

**Figure 9:**
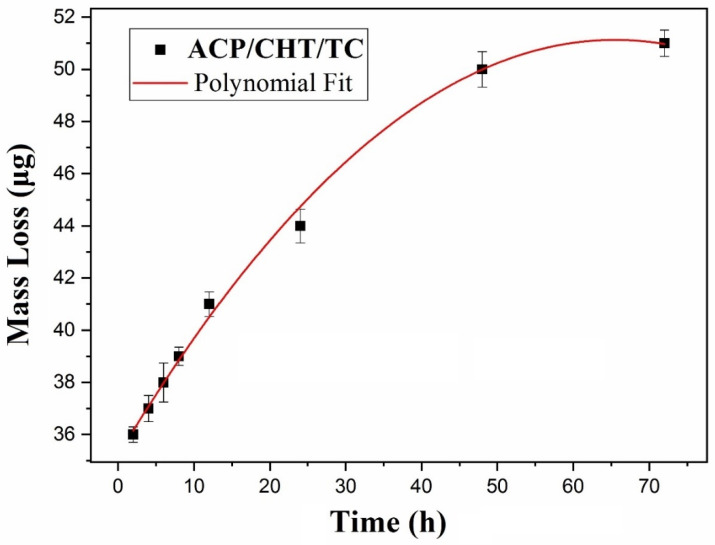
Mass Loss Release, Δ*m*, as a function of immersion time in SBF, *t*.

The composite film ACP/CHT/TC is hydrophilic: 61°/11° and 53 mN/m. The hydrophilicity increase trend is reflected by the CA decrease from (89°/47.8°, 35.5 mN/m) to (61°/11°, 53 mN/m), i.e., values that are even slightly inferior to those of ACP (Figure 9). This can prove beneficial for coating “endorsement” by the neighbor tissues in the body and the protected growth of bone cells.

### 3.2. Composite Mixture (Containing Drug) Delivery

The degradation and drug release kinetic was evaluated by mass loss measurements, UV-Vis, and pH studies.

#### 3.2.1. Mass Loss

The mass, *M*_0_, of as-deposited composite film was measured for 16 samples. An average weight of deposited films of 71 ± 7 μg was inferred, with a SD of ±10%, which is due to both the inherent mass variation between MAPLE samples deposited under identical conditions and the accuracy of the weighting measurements.

The mass loss from film, Δ*m*(*t*), was estimated with Equation (1). Measurements were conducted for *t* (immersion duration in SBF) of 2, 4, 6, 8, 12, 24, 48, and 72 h, respectively, and the results were represented in Figure 9.

A continuous increasing trend of Δ*m*(*t*) is noticed from Figure 9, with a saturation tendency towards longer immersion durations. The experimental data were fitted with a polynomial law of the lowest order, in this case quadratic:

Δ*m*(*t*) = *A*_0_ + *B*_0_*t + *C*_0_*t^2^ (3)

for 2 ≤ *t* ≤ 72, in [h]. The best fitting equation was obtained for *A*_0_ = 35.3 [μg], *B*_0_ = 0.47 [μg/h] and *C*_0_ = −0.0035 [μg/h^2^], (R^2^ = 0.997).

This law can serve to predict the time behavior of such composite mixture deposits in SBF, and by extension in BF (actual body fluids), i.e., in contact with living tissues.

It is obvious from Figure 9 that antibiotic delivery included in the composite mixture lasts for long time, much longer than 6 h, a fact that is usually associated with TC release when administrated orally or by injection. In particular, from the 6th to 72nd h, about (20 ± 2)% of the initial mass continued to be released in SBF (relative delivery rate, *p*, as calculated with Equation (2)). The delivery process continued even further, as about (27 ± 2.7)% of the film was still on the surface after 72 h of immersion.

#### 3.2.2. UV-Vis Studies

**Figure 10:**
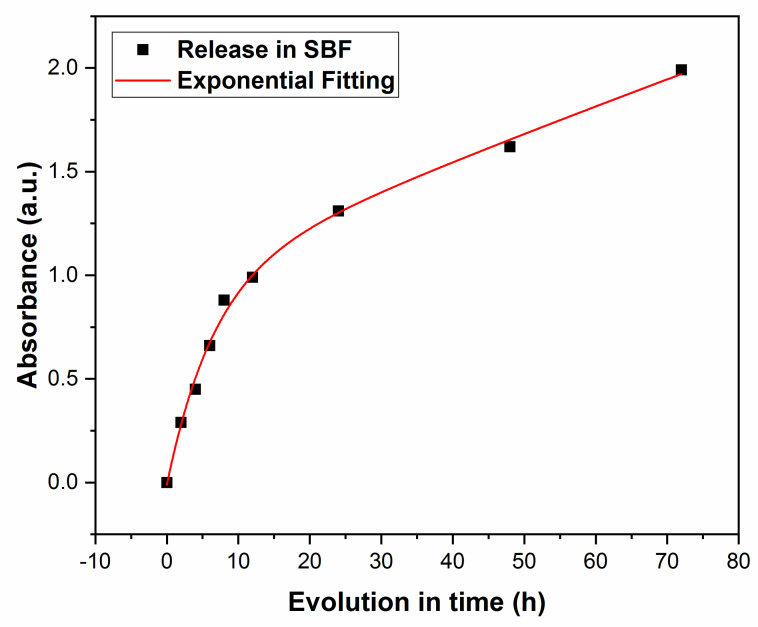
Time evolution of the solution’s absorbance.

Next, drug release was monitored by measuring the solution’s absorbance, *A*(*t*), at λ = 201 nm, after different immersion times, with *t* in h. One notices from Figure 10 an increasing trend in the absorbance along the immersion time, *A*(*t*), with a gradual saturation towards longer durations. The evolution of *A*(*t*) is congruent with that of Δ*m*(*t*) in Figure 9, demonstrating that the substance released from the film stays at the origin of the solution’s absorbance evolution in time.

The experimental data were fitted with an exponential law of the type:

*A*(*t*) = *A*_2_ + *B*_2_e*^αt^* (4)

for 2 ≤ *t* ≤ 72, in [h]. Here, *A*_2_ is the intercept; *B*_2_ is the slot, while *α* is the increment of the composite release in the solution; *t* is the time in *h*. In this case, the best fit was obtained for *A*_2_ = 1.90; *B*_2_ = −1.76 and *α* = −0.05 [h^−1^] (R^2^ = 0.968).

#### 3.2.3. pH Measurements

**Figure 11:**
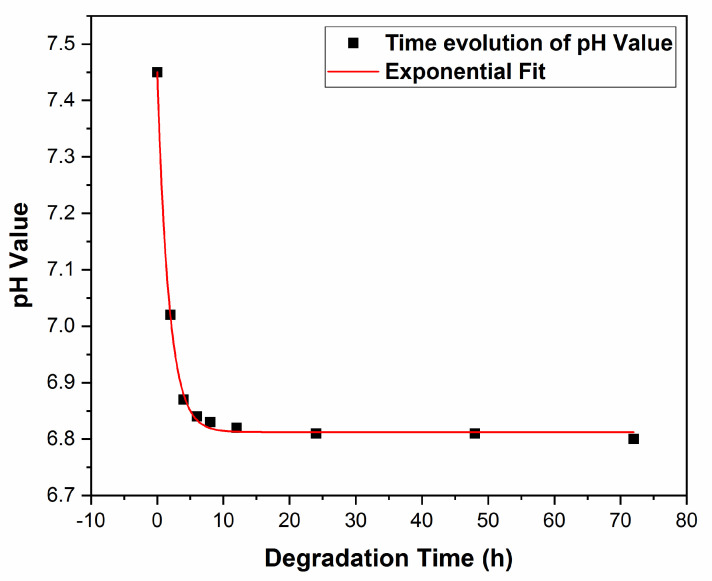
pH evolution with immersion time in SBF.

pH behavior after degradation in SBF was represented in Figure 11.

One observes an exponential decrease in time, as opposed to absorbance, *A*(*t*) (see Figure 10). The best fit of the experimental data was obtained, in this case, with an exponential law of the type:

*pH*(*t*) = *A*_3_ + *B*_3_e^*βt*^ (5)

for 2 ≤ *t* ≤ 72, in [h], where: *A*_3_ = 6.81; *B*_3_ = 0.633 and *β* = 1.76 [h^−1^] (R^2^ = 0.99).

For *β*, the increment of pH is in this case positive, as contrasted with the coefficient α, in Equation (4), which is negative.

As is visible from Figure 11, the initial pH value corresponding to pure SBF is 7.45, i.e., very close to the physiological optimum value in the range (7.35–7.45) [65]. Next, pH slowly decreases with the composite concentration increase, to stabilize at about 6.8, for immersion durations *t* ≤ 72 h.

One should mention that these small pH shifts of 0.55 and 0.65, respectively, toward a more acidic medium can have positive effects. Indeed, it can stimulate the osteoclast’s generation derived from the bone marrow precursor’s cells and enhance osteoclastic resorption [70]. Also, as pointed out in [70], acidosis plays a key role in activation via up-regulating various genes responsible for cell adhesion, migration, survival, and bone matrix degradation.

### 3.3. Microbiological Assays

#### 3.3.1. Cellular Morphology

The microscopic results were interpreted in accordance with the recommendations of ISO 10993-5:2009(E); Part 5: “Biological evaluation of medical devices. In vitro cytotoxicity tests”.

**Figure 12:**
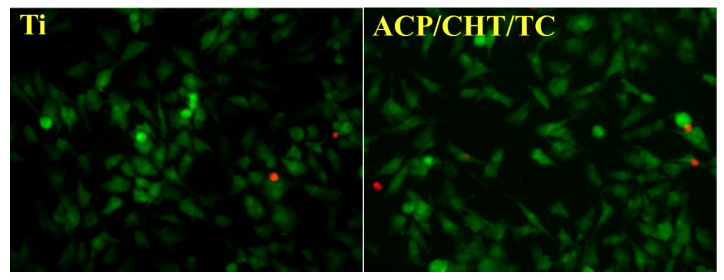
Fluorescence microscopy images of MG63 cells grown after 24 h on as-deposited composite films (**right**) and on Ti control disks (**left**), respectively. Staining with PI and FDA (magnification ×200).

One notes from Figure 12 that the morphology growth of MG63 cells on films or Ti control is similar. No changes in cellular morphology were observed that could be indicative for the advent of a majority of round, contracted dead cells.

#### 3.3.2. Cellular Cycle Assay

**Figure 13:**
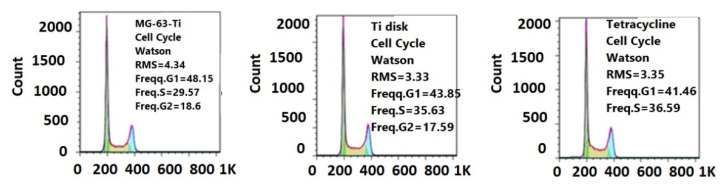
Flow cytometry diagrams of cellular cycle of MG63 cells grown on a standard plastic substrate (**left**), Ti control disk (**center**), and deposited composite film (**right**).

The results by microscopic examination were confirmed by the flow cytometry assay of the cellular cycle, showing no changes in the distribution of growing phases (Figure 13), confirming the biocompatible, non-cytotoxic character of deposited films.

#### 3.3.3. Antimicrobial Biofilm Activity

**Figure 14:**
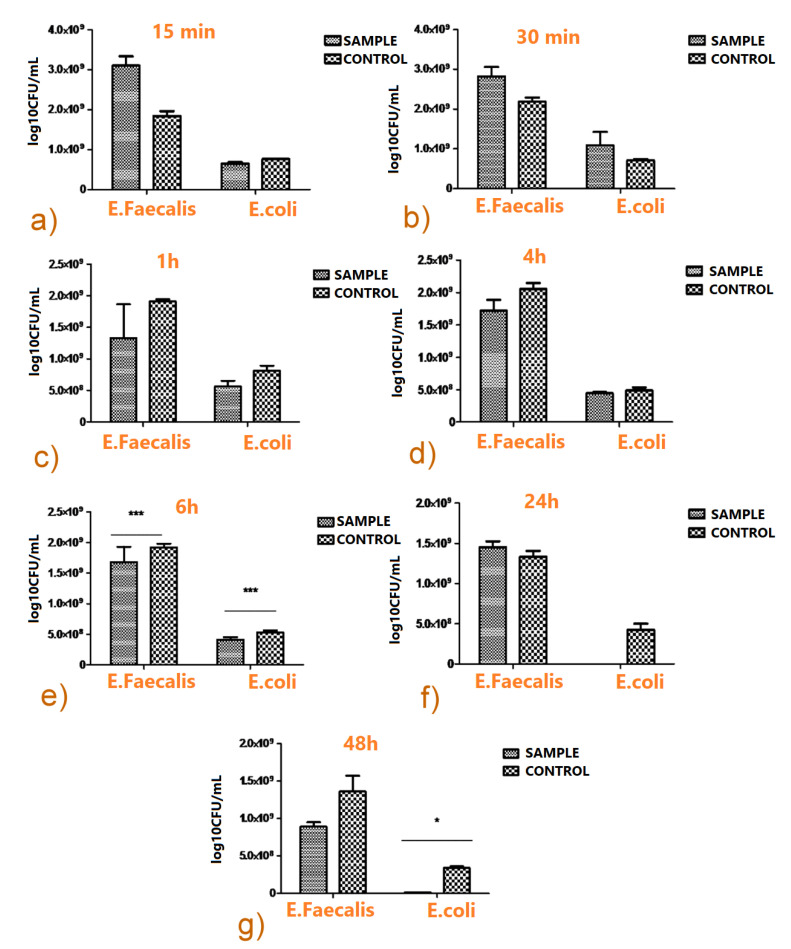
Microbial growth dynamics on as-deposited composite films vs. Ti control after 15 min (**a**), 30 min (**b**), 1 h (**c**), 4 h (**d**), 6 h (**e**), 24 h (**f**), and 48 h (**g**) incubation time, respectively (two-way ANOVA Bonferroni test, * *p* < 0.05; *** *p* < 0.001).

In order to quantify both the initial fast release of the antibiotic in active form, as well as the duration of the drug protective action, the antimicrobial activity was assessed within a temporal “window” extending from 15 min to 48 h. No inhibitory effects were detected for deposited films as compared to the Ti control, in the case of the first two incubation times of 15 and 30 min (Figure 14a,b).

The inhibitory effect of TC-containing films becomes more significant starting with the first hour of incubation, as compared to the Ti control. The number of VCCs recovered from functionalized films was inferior to the control for the two bacterial strains tested (Figure 14c).

The anti-biofilm effect was preserved after 4 (Figure 14d) and 6 h (Figure 14e) for all bacterial strains. The differences between thin films and the Ti control became statistically relevant in the case of *E. faecalis* and *E. coli* biofilms developed after 6 h of incubation.

The inhibitory effect of composite thin films was maintained after 24 h for *E. coli* only (Figure 14f). An important anti-biofilm effect of composite layers with more than one log decrease in the VCCs was noticed after 48 h for both *E. faecalis* and *E. coli* strains (Figure 14g).

## 4. Discussion

We fabricated, using the MAPLE technique, structures with double functionality: mechanical and biological, for long-time action against the contamination of implant coatings and prevention of nosocomial and other large area infections. Special care was paid to avoid resistance to antibiotics. Our solution was to apply the antibiotic, TC, in a mixture with ACP and CHT. The two additives were introduced for extended storage of TC and prolonged release in time to the surrounding tissues (via fluids), to achieve maximum effects under any particular configuration.

SEM observation of as-deposited films evidenced rough surfaces, uniformly covering the substrates. By contrast, the SEM examination after 72 h immersion in SBF revealed a “cavernous” structure from which a notable amount of composite material was extracted and delivered into the solution. This evidence is in accordance with the mechanism of controlled drug delivery from porous structures, described in Reference [45].

FTIR results indicated the congruent transfer of the substance from the composite target to the MAPLE films. The substance still present on the substrate after biodegradation is chemically similar to the raw powders and as-deposited films.

As-deposited films were generally amorphous, as shown by XRD investigations. After degradation, the XRD diffractogram revealed the same predominant amorphous appearance.

Wettability studies confirmed the sponge-like nature of as-deposited films, in good accordance with SEM and XRD data, sustaining an overall amorphous structure. One important note concerns the large decrease in the CA, from ~89° in the case of as-deposited film to only ~60° after 72 h immersion in SBF. This could be the effect of the amorphous nature of coatings (see Figure 6) where liquids are more easily trapped, as reflected by the increase in the degree of hydrophilicity. This evolution is favorable to nosocomial and large area protection applications of as-deposited structures, while the consistently smaller value after biodegradation is benefic for cell activation, differentiation, and, finally, spreading over implantable devices. Our studies revealed that hydrophilicity increases with the TC inclusion into the composite mixture, which supports the hydrophilic nature of this drug. It is well known that the materials’ abilities to attract water or other liquid molecules (described as hydrophilicity) stands for a key factor in cell adhesion and osteogenesis regulation [59]. Small CA values imply good wettability, involving large surface energies [36,55].

Mass loss monitoring from deposited films exhibited an initial rapid release (during the first 6 h), followed by a slow and constant tail. Thus, the relative mass loss (Δ*m*/*m*_0_) jumps to 57% after 6 h, to stabilize to 62, 70, and 73% after the first, second, and third days of immersion in SBF, respectively. This behavior is intermediated, in our opinion, by the TC storage and time conservancy in ACP and CHT. It proves that our approach opens a new alternative to conventional systemic treatments and overcomes the burst release of TC small molecules. By loading TC in a proper carrier (in our case, the two other ingredients of the mixture), “the burst” drug release gets slowed down and is under control, meanwhile ensuring a prolonged and efficient delivery of the drug.

On the other hand, the slow, quasi-constant release of the substance could presumably involve the degradation of the composite matrix, combined with the chemical desorption or diffusion of TC. The relative mass loss released after 72 h (of about 73%) could be thus indicative for TC bonding with the composite matrix. This evolution was further supported by the UV–Vis spectroscopy quantitative analysis. As expected, the experimental calibration curve, *A*(*c*), was linear (Figure S2 Supplementary Material), pointing to a direct proportionality between absorbance and composite mixture concentration in solution. The same applies to the inverse calibration law, *c*(*A*), as deduced by simple calculation from Equation (4) for *A*(*c*), which reads as:

*c*(*A*) = *A*_5_ + *B*_5_*A (6)

Here, *A*_5_ = 4 μg/mL and *B*_5_ = 66.7 μg/mL, for *A* ≤ 2, [arb. units].

**Figure 15:**
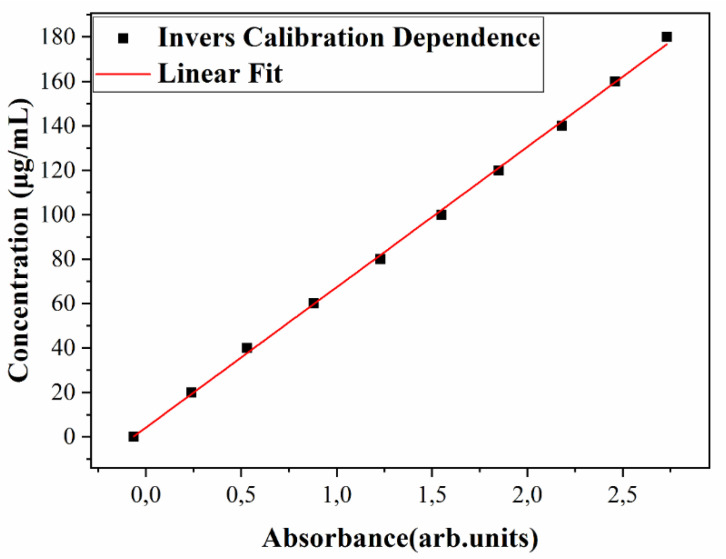
Inverse calibration dependence, *c*(*A*).

The inverse calibration law, Equation (6), was graphically represented in Figure 15, wherefrom one notices a variation in *c* from 20 to 140 μg/mL, i.e., within the range actually used for the preparation of diluted solutions.

By comparison of Equations (4) and (6), one observes a higher slope (*B*) in the second case (*B*_5_ > *B*_2_), which allows for an improved discrimination of the experimental data on this curve.

Next, the experimental time evolution of absorbance, *A*(*t*) (Figure 10) was the best fitted by an exponential law with a negative increment, *α* = −0.05. One observes from Figure 10 a rapid initial increase in absorbance, up to 0.66 after 6 h, followed by saturation to 1.31 after the first, 1.62 after the second, and 1.99 after the third day of immersion, respectively.

From Equations (5) and (6), the time evolution of the composite concentration in the solution can be inferred as:

*c*(*t*) = *A*_6_ + *B*_6_ * e^*αt*^ (7)

where *A*_6_ = 131 μg/mL, *B*_6_ = 117 μg/mL and α = −0.05 for 2 ≤ *t* ≤ 72 [h].

**Figure 16:**
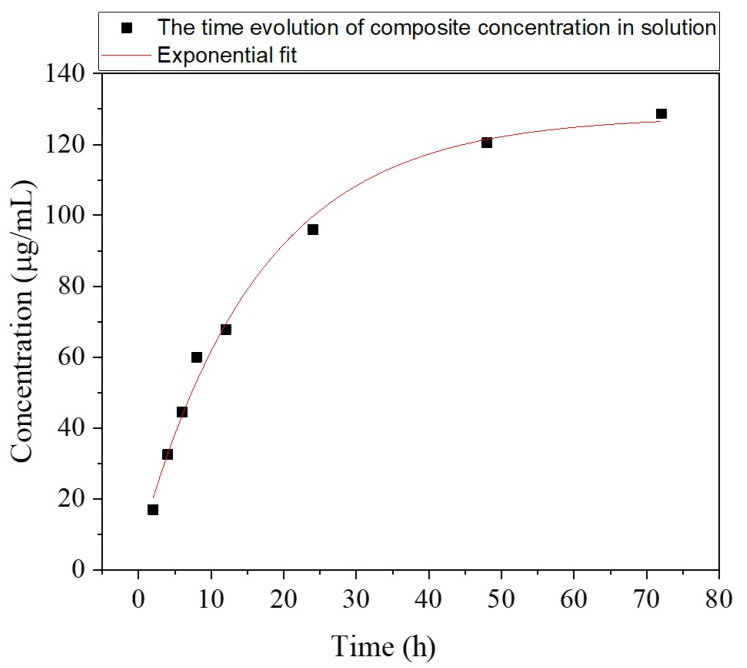
The time evolution of composite concentration in solution, *c*(*t*).

Equation (7) was graphically represented in Figure 16, from which one observes that *c*(*t*) reaches 44.5 μg/mL after 6 h, then increases to 67.8 after 12 h, 96 after 24 h, 120.47 after 48 h, and 128.66 after 72 h, respectively. One important remark is that all these values are confined within the concentration range used for the preparation of diluted solutions in our experiments (≤180 μg/mL).

This evolution is in accordance with the aforementioned anti-biofilm action of the composite, that exhibited a very strong decrease in VCCs after 48 h for both *E. faecalis* and *E. coli* strains.

At the same time, an exponential decrease in time was inferred for the pH solution, with a very low quasistatic decrease slope at longer durations. The effect was a pH variation of only 0.64 after 24 h, as well as after 48 and 72 h. This was able to produce only a very limited increase in medium acidity of about 8% in respect to the initial value, which is insufficient to initiate significant perturbation (apoptosis) of MG63 fibroblast cells. This small shift does not influence the normal growth of MG63 cells, which remain practically unperturbed after 12 h and for longer incubation times. It should be mentioned that the local acidity plays a role in early wound healing, infections, and bone remodeling. The drug release kinetic observed was shown to be a combination of the composite matrix degradation and drug diffusion. According to mass loss data, the matrix degradation seems to drive the release, whilst the progressive diffusion/desorption becomes predominant at the end of the release.

This results in the obtained biocompatible and osteoconductive drug delivery carriers tuning the deposited film degradation via adjustment of the release mechanism of TC from coatings. An optimal balance between biodegradability and bioactivity could be reached by the proper selection of mixture composition, so as to ensure an osteoconductive surface for cell adhesion and proliferation.

## 5. Conclusions

We report on the MAPLE deposition of composite thin layers for long-term protection of surfaces against microbial infections. A multidrug combination therapy was proposed for local and sustained delivery with antimicrobial action, while concurrently inhibiting the drug’s resistance mechanisms and promoting bone regeneration and growth.

A composite mixture consisting of ACP-CHT-TC in deionized water was used for preparation of the MAPLE solutions, that were frozen in LN to get icy MAPLE targets. Thus, a chemical composition similar to natural bone is expected by combining the natural chitosan (organic part) with amorphous calcium phosphate (inorganic part). Tetracycline coupling will further promote and prolong the antimicrobial efficacy. This way, the risks of overdose, frequent systemic administration, and side effects could be significantly reduced.

As-deposited films were compact and covered with micron-size particulates. The scavenger character of coatings was supported by XRD results pointing to a prevalent amorphous structure. FTIR studies confirmed the general congruent transfer of the substance from the target to the deposition side.

Contact angle measurements revealed a wettability degree of as-deposited films at the border between hydrophilicity and hydrophobicity. The films were strongly converted to the hydrophilicity side after immersion in the SBF. This behavior supports the use of as-deposited structures against nosocomial infections and large area protection, and the efficient application of degraded films as friendly implant coatings.

Direct and inverse calibration laws relating solution absorbance, *A*, and dissolved composite concentration, *c*, were pointing to a direct proportionality between the two parameters. The time evolution of both *A* and *c* was exponential, with an increment describing a first fast increase (burst) followed by a saturated long tail.

This explains why the antimicrobial agents in the composite mixture stay active after quite a long immersion time (up to three days) as demonstrated by the efficient action against *E. faecalis* and *E. coli* strains.

An exponential decrease in the pH of a solution with composite was inferred. A first pH decrease from 7.45 followed by a long, stable tail at 6.8 was thus observed. This is, in our opinion, responsible for the quite limited action of the composite solution against MG63 fibroblast cells, pointing to an overall biocompatible behavior of deposited structures.

One may conclude that the composite mixture in deposited films exhibits increased antimicrobial properties, excellent biocompatibility, superior mechanical parameters, and extended drug release over time, from a few hours to a few days. This coFigntrolled release represents the main advantage of an in situ administration of TC, together with lower consumption of excipients, decreased doses of antibiotics, and, hence, lower metabolic residues and reduced rates of local infection and repetitive, costly surgery. The MAPLE method opens new frontiers in smart implant coatings and anti-pollutants for nosocomial and other large surface areas’ protection.

## Data Availability

Not applicable.

## Supplementary Materials

The following are available online at https://www.mdpi.com/article/10.3390/pharmaceutics13101659/s1, Figure S1: Supplementary material: EDS data of the composite deposited film after 72 h of degradation. Figure S2: Calibration curve.
