# Supplementary material for: Composite Drug Delivery System Based on Amorphous Calcium Phosphate–Chitosan: An Efficient Antimicrobial Platform for Extended Release of Tetracycline
Source: Pharmaceutics. 2021 Oct 11;13(10):1659. doi: 10.3390/pharmaceutics13101659 (PMC8537227; doi:10.3390/pharmaceutics13101659)
Supplement: Supplementary file 1 [file pharmaceutics-13-01659-s001.zip › pharmaceutics-1387468-supplementary.pdf]

pharmaceutics

MDPI

Supplementary Materials: Composite Drug Delivery System Based on Amorphous Calcium Phosphate–Chitosan: An Efficient Antimicrobial Platform for Extended Release of Tetracycline

Anita Ioana Visan, Carmen Ristoscu, Gianina Popescu-Pelin, Mihai Sopronyi, Consuela Elena Matei, Gabriel Socol, Mariana Carmen Chifiriuc, Coralia Bleotu, David Grossin, Fabien Brouillet, Sylvain Le Grill, Ghislaine Bertrand, Irina Zgura, Rodica Cristescu, Ion N. Mihailescu

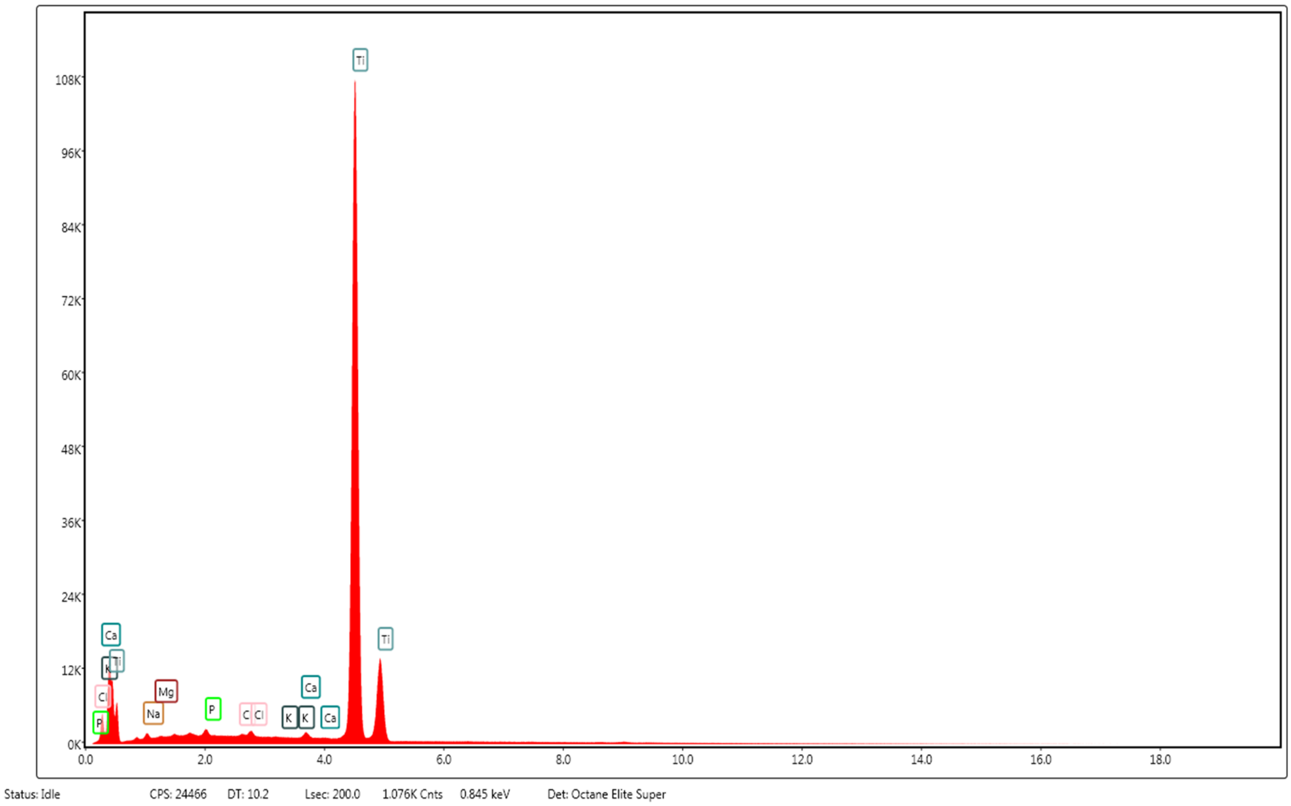

Figure S1. Supplementary material: EDS data of the composite deposited film after 72 h of degradation.

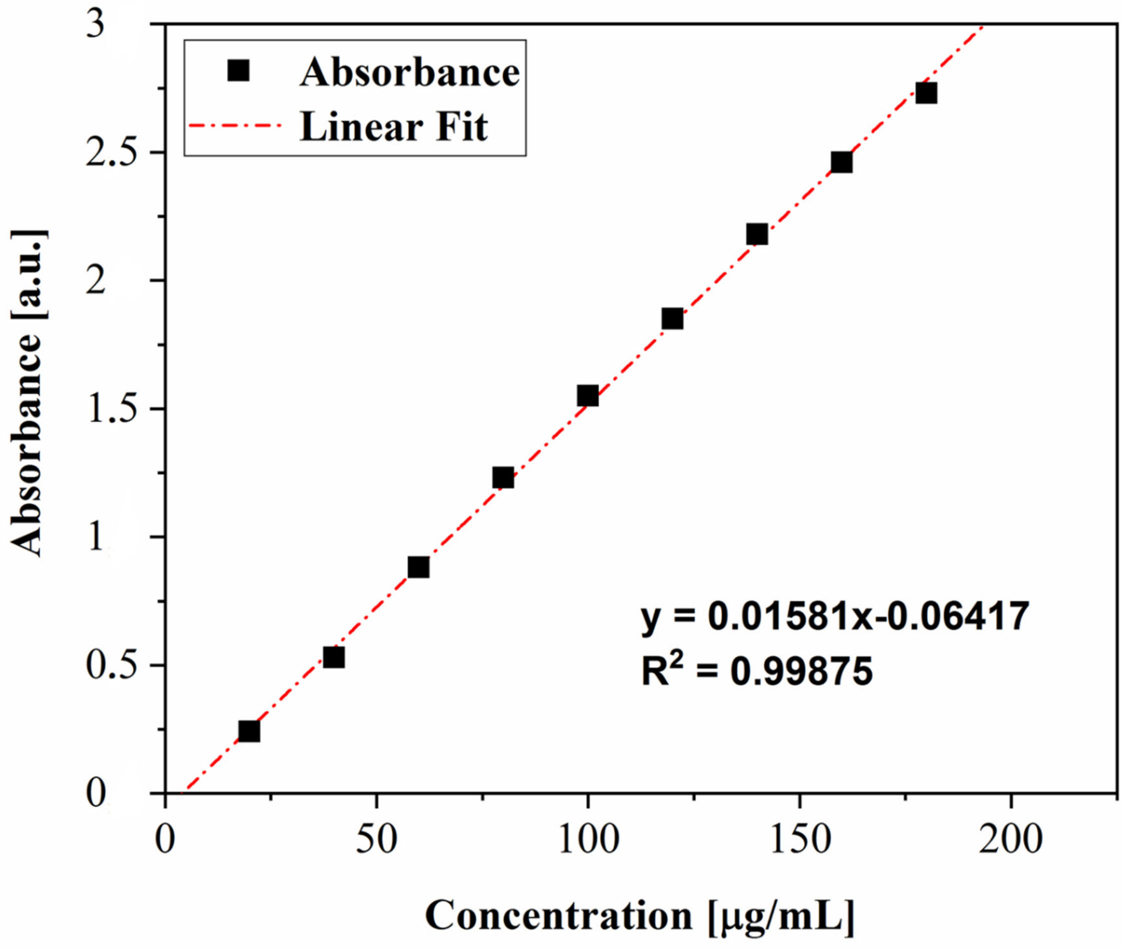

Figure S2. Calibration curve.
